# The association between historical childhood sexual abuse and later parenting stress: a systematic review

**DOI:** 10.1007/s00737-016-0708-3

**Published:** 2017-01-04

**Authors:** Melanie Hugill, Katherine Berry, Ian Fletcher

**Affiliations:** 1 Department of Health Research, Faculty of Health and Medicine, Furness College, Lancaster University, Lancaster, LA1 4YG UK; 2 Division of Psychology and Mental Health, School of Health Sciences, University of Manchester, Zochonis Building, Brunswick Street, Manchester, M13 9PT UK

**Keywords:** Childhood sexual abuse (CSA), Parenting stress, Systematic review

## Abstract

An individual’s own experiences of childhood and being parented are likely to be key determinants of their later parenting experiences. Childhood sexual abuse (CSA) is arguably the most toxic experience to occur in childhood and therefore may be particularly likely to impact on parenting stress in the context of parenting one’s own children. This paper aims to review studies investigating associations between earlier CSA and later parenting to determine the size and consistency of the effects, identify any mediators and moderators of the relationship, and assess the quality of the evidence base. PsycINFO, Academic Search Complete, CINAHL, MEDLINE, Web of Science, PubMed and PILOTS were searched from date of inception until 4th March 2016 and 14 studies met the inclusion criteria. Seven studies indicated a degree of direct association between experiencing CSA and later parenting stress, two studies found no association and five studies suggest that other variables such as locus of control and current stressors may affect the relationship between CSA and parenting stress. Additionally, 10 studies suggest an indirect relationship between CSA and parenting stress through current level of depression. Results suggest the existence of a relationship between CSA and parenting stress though this association is mostly mediated by other variables, including depression and other stressors. Clearer definitions of CSA and use of validated questionnaires are essential to progress this field of research.

## Introduction

It is widely acknowledged that parenting, and first-time parenting in particular, may be stressful. Parenting stress can be defined as “the aversive psychological reaction to the demands of being a parent” (Deater-Deckard 1998 p. 315). However, this reaction is multi-faceted and relies on several factors including (and not limited to) the parents’ psychological health, their relationship with their child, sources of support and their own experiences of being parented (Anthony et al. 2005). Parents will therefore differ in terms of the amount of stress they experience, though it is expected that most parents will experience stress at some point. Research suggests that elevated parental stress can have a negative effect on the parent–child relationship (Deater-Deckard and Scarr 1996). For instance, stress can intensify harsh and more punitive parenting styles, resulting in lower emotional well-being for children (Crnic et al. 2005). Behavioural problems may also be exacerbated by such parenting which may increase levels of parenting stress, indicating the existence of a bidirectional relationship (Vallotton et al. 2016).

An individual’s own experiences of childhood and being parented are likely to be key determinants of their parenting style. Research has demonstrated that childhood maltreatment experiences are likely to have detrimental effects on subsequent parenting abilities (Fitzgerald et al. 2005). For instance, a robust association was identified between mothers who had experienced childhood physical abuse and records of maltreatment of their infants before the age of 26 months (Berlin et al. 2011). Mothers who experienced childhood emotional abuse have been reported to display reduced empathic responding to their six-month old infants and score lower on measures of parental self-efficacy (Bert et al. 2009; Caldwell et al. 2011). Additionally, the early experience of CSA has been associated with more permissive practises in later parenting and an increased potential for the abuse or neglect of offspring (Ruscio 2001; Trickett et al. 2011). Such evidence suggests that difficult childhood experiences may have pervasive and enduring consequences which affects an individual’s relational style throughout life, including in the parenting role.

With regard to CSA, it is widely recognised that the experience of CSA can be detrimental both to the developing child and later in life (Wohab and Akhter 2010). Recent research has also highlighted that CSA may affect the structure and function of some areas of the brain, including the hippocampus, amygdala and cerebral cortex (Teicher and Samson 2016). While a thorough review of this research is beyond the scope of this paper, the emerging picture is that these structural and functional changes as a result of CSA may make the individual more vulnerable to later stress and affect their ability to cope with this stress. It is therefore not surprising that CSA is associated with psychopathology in adulthood, including depression, psychotic symptoms and substance abuse (Coles et al. 2015).

A number of studies have now investigated how CSA affects parenting abilities including parenting stress, though to date no systematic review has been conducted looking specifically at CSA and later parenting stress. This paper aims to review these studies to determine the consistency and size of effects, and the quality of the literature. Furthermore, a review will highlight other important factors that may moderate or mediate this relationship. Understanding factors that moderate the relationship between CSA and parenting stress is important as these variables may affect the strength of this relationship. For example, more severe types of CSA such as incest have been associated with the most severe and long-reaching effects (Essabar et al. 2015), though it is not known whether these factors, or indeed any other moderators, are important with regard to parenting stress.

It is also important to determine if any mediating variables are indicated in the relationship between CSA and parenting stress, as mediators explain the underlying mechanisms via which one variable affects another. For instance, there is an established link between parenting stress and depression, particularly in the postnatal period (Epifanio et al. 2015) and research has also suggested a possible link between Postpartum Depression (PPD) and historical childhood sexual abuse (Wosu et al. 2015). This suggests that depression may mediate the relationship between CSA and later parenting stress. Identifying mediating variables is important as these may provide opportunities to intervene in the relationship between CSA and parenting stress.

Previous reviews on parenting practises of adult CSA survivors contain limited reference to parenting stress. An early paper by DiLillo and Damashek (2003) reviewed the parenting characteristics of CSA survivors, but this review only included two studies which had used a measure of parenting stress; one of which suggested no association between CSA and parenting stress (Alexander et al. 2000) and one which suggested mothers with a history of CSA reported elevated stress compared to controls (Douglas 2000). A more recent review by De Jong et al. (2015) on the transition to adulthood of CSA victims also cites the Douglas (2000) paper which indicated a significant association between CSA and parenting stress, but cites no further studies regarding parenting stress. However, De Jong et al. included only contact abuse studies in their review, excluding studies that reported both contact and non-contact abuse together, and furthermore only included studies which used a non-abused comparison group. This means that a number of studies may have been omitted and the results they report are therefore limited and not representative of the range of experiences of CSA survivors.

In summary, the increasing awareness of the negative sequelae caused by stress both on the parent–child relationship and on the developing child means an understanding of factors that increase parenting stress is vital. Therefore, the aims of this systematic review are to examine the literature to determine the consistency and strength of association between CSA and later parenting stress and to assess the quality of the studies found. Any mediators or moderators between CSA and parenting stress will also be explored.

### Method

To ensure clarity of reporting this systematic review has been conducted in accordance with the Preferred Reporting Items for Systematic Reviews and Meta-Analyses (PRISMA) Statement (Liberati et al. 2009). The inclusion criteria for this systematic review were as follows: 1) participants who had experienced historical CSA and were now parents, 2) a self-report measure of stress; 3) English language and 4) published in a peer-reviewed journal. Studies which did not separate CSA from other types of childhood maltreatment were excluded. No restrictions were placed on the age of participants or on date of publication. Potential studies were identified by searching electronic databases between 14th January and 4th March 2016. The following databases were searched from date of inception until 4th March 2016: PsycINFO, Academic Search Complete, CINAHL, MEDLINE, Web of Science, PubMed and PILOTS. Each database was searched individually using the same key words and any specific thesaurus/MeSH headings suggested by the database. Additionally, reference lists of potential articles were hand searched and Google Scholar was used to perform citation searches on these potential articles. Search terms were selected from reviewing literature pertaining to CSA and parenting stress and in particular search terms used in previous systematic reviews of CSA, for example Wosu et al. (2015). The following terms were used in each database: parent* OR maternal OR paternal OR mother OR father AND stress* OR distress* AND earl* OR surviv* OR childhood OR previous OR prior AND abus* OR trauma* OR maltreat* OR advers*. Individual database thesaurus terms were also used to ensure no studies were missed.

**Fig. 1:**
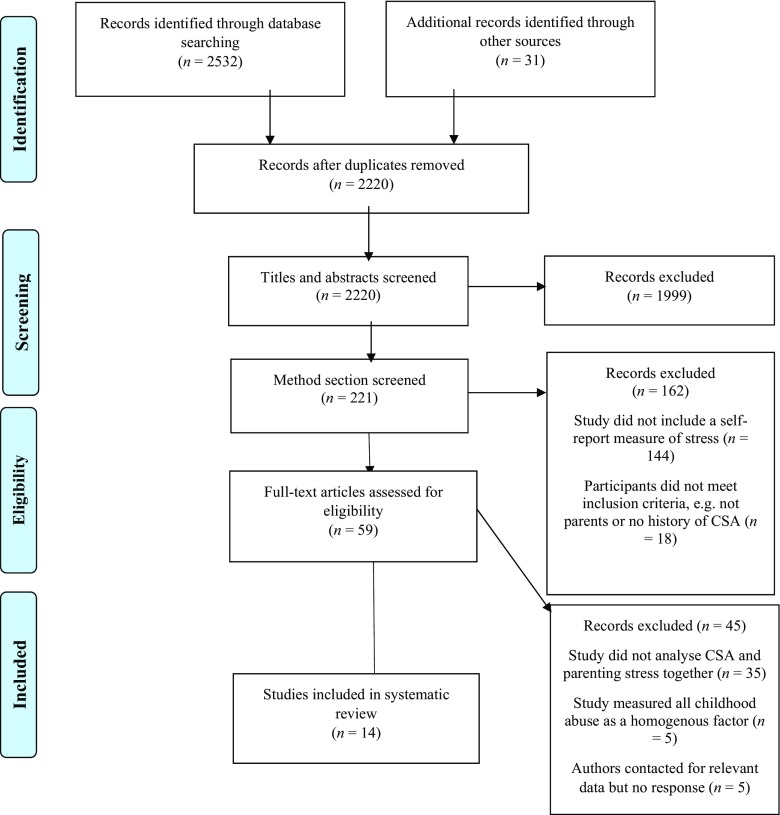
PRISMA flow diagram depicting study selection

The PRISMA flow diagram is presented in Fig. 1 to summarise the study selection and screening process. Studies identified in each database search were transferred to EndNote to allow removal of duplicates. Following this, 2220 titles and abstracts were screened for eligibility, which led to the exclusion of 1999. The method sections of the remaining 221 records were then screened leading to the exclusion of 162. The main reason for exclusion at this stage being the absence of a self-report measure of parenting stress. The full text of the remaining 59 studies was reviewed and a further 45 excluded, the reasons for which are: (a) the study did not report the analysis between CSA and parenting stress (*n* = 35) and (b) the study measured all childhood abuse as a homogenous factor (*n* = 5). Finally, five study authors were contacted for data necessary to facilitate inclusion in the review. These studies had used measures appropriate for inclusion in the review, but the article did not report the analysis between these measures. However, the authors did not respond so the studies could not be included. This left 14 studies for inclusion in the systematic review. Throughout the screening process any papers which the first author was unsure about including were discussed and agreed with the research team.

Data was extracted from each study on (a) study design and participant characteristics (including study design, country of origin, ethnicity of sample, number of participants, type of sample and mean age of parent), and (b) the measures used for CSA and parenting stress, the type of analysis used and the results obtained. Table 1 presents the study characteristics and demographic data for the participants in each study and Table 2 presents the measures used in each study, how the data was analysed and the results from each study.

**Table 1:** Demographic information from studies

Study	Study design	Country	Ethnicity	Participants	Type of sample	Mean age of mother
Alexander et al. (2000)	Cross-sectional	USA	80% Caucasian, 11.1% African-American, 8.9% Latina	90 mothers (19 (21%) reported CSA)	Community - response to advert	36.4
Barrett (2009)	Secondary data analysis from a panel (longitudinal) study	USA	Predominately African-American (82.7%)	483 mothers (54 (11%) reported CSA)	Community – benefit recipients	28.83
Buist and Janson (2001)	3 year prospective study	Australia	Not reported	45 mothers who had developed depression postpartum (23 (51%) reported CSA)	Clinical - mother & baby unit. Mothers diagnosed with either major depression or adjustment disorder	CSA group: 30.5 Control: 31.6
Douglas (2000)	Case–control	Scotland	Not reported	63 mothers (34 (54%) reported CSA)	Clinical – mental health out-patient clinic	CSA group: 31.7 Control: 35.8
Ethier et al. (1995)	Case–control	Canada (French speaking)	Not reported	80 mothers (40 “negligent” & 40 control). Frequency of sexual abuse “events” reported: 20 events reported in the negligent group, 14 in the control	Neglecting mothers from Youth Protection Services, matched with controls from the community	28.6 (neglecting) 30.3 (control)
Harmer et al. (1999)	Cross-sectional	Australia	Predominately Anglo-Saxon, three mothers identified themselves as half native aboriginal	46 mothers recovering from drug or alcohol addiction (22 out of 39 (56%) who completed the CSA measure reported CSA)	Clinical (recovering addicts) residing at a therapeutic community	28.5
Lang et al. (2010)	Prospective: Time point 1 in early pregnancy, time point two when child was 12 months old	USA	61.4% Caucasian, 18.2% Hispanic, 11.4% African-American, 9.1% “other”	44 mothers at time point one, 31 at time point two (70.4%). 20.4% reported moderate/severe CSA at time point one	Community - response to advert	29.27
Lutenbacher (2000)	Cross-sectional	USA	56% African-American, no other information given	59 low income mothers (9 reported CSA only, 11 (19%) reported a mixture of CSA and physical abuse)	Community - response to advert and approached by staff	26.1
Mapp (2006)^a^	Secondary data analysis from a cross-sectional prospective study between 1991 and 1998 (used data only from time point 2)	USA	73% African-American, no other information given	265 (40.4% reported CSA)	Community – from a prenatal clinic	Not reported
Pazdera et al. (2013)^a^	Secondary data analysis from a cross-sectional prospective study between 1991 and 1998	USA	73% African-American, no other information given	265 mothers (number of CSA survivors not reported, but assumed to be 40.4% as above)	Community – from a prenatal clinic	Not reported
Pereira et al. (2012)	Cross-sectional	Canada	67.2% Caucasian, 13.2% Asian, 5.6% Hispanic, 3.8% mixed ethnicity, 2.8% African, 0.6% North American, 3.1% “other”	291 mothers (50 (17%) reported CSA)	Community - response to advert and approached by staff	33.38
Renner et al. (2015)^a^	Secondary data analysis from a cross-sectional prospective study between 1991 and 1998	USA	73% African-American	264 mothers (107 (40.5%) CSA survivors), 1 excluded for excessive missing data	Community – from a prenatal clinic	26.98
Schuetze and Eiden (2005)^a^	Secondary data analysis from a cross-sectional prospective study between 1991 and 1998	USA	73% African-American, 27% Caucasian	263 mothers (107 (40.6%) reported CSA)	Community – from a prenatal clinic	26.99
Wright et al. (2005)	Cross-sectional	USA	96% Caucasian	79 mothers (all self-reported CSA)	Community - response to advert	38.2

^a^Same primary data set used

**Table 2:** The measures used in each study, the type of data analysis, main results from each study and global quality rating score

Study	Measure of CSA	Measure of parental stress	Analysis	Result	Global quality score
Alexander et al. (2000)	Questions: When you were a child or adolescent, did anyone ever actually touch private parts of your body or make you touch theirs against your wishes or when you were asleep, drugged or in some other way helpless? Further questions were asked re age, frequency etc. if answered yes to above	PSI-SF	Analysis of covariance – main & interactive effects of CSA & relationship satisfaction on parenting stress.	No main effect of CSA on parenting stress (no figures provided).	Weak
Barrett (2009)	2 questions: 1) Has a stranger, acquaintance, date or relative ever tried or succeeded in doing something sexual to you against your wishes? 2) How old were you the first time this happened?	A scale taken from a women’s employment study which included items from the PSI	T-tests: Hierarchal multiple regression:	CSA mean stress score x Control mean stress score *t*(481) = −1.02, *p* = .38 (not sig). Cohen’s *d* = .15. *β* = −.01, *p* = .90 (*ns*).	Moderate
Buist and Janson (2001)	Abbreviated version of the Otago Women’s Health Survey (Martin, Anderson, Roman, & O’Shea, 1993). Asks details of the abuse, age of onset, age and gender of perpetrator, relationship to perpetrator and whether the victim confided in anyone at the time, regarding the abuse.	PSI	Two sample t-tests	Means for index and comparison groups not significantly different on either parent or child domain of PSI **BUT** life stress subscale on PSI significantly higher in the index group (*p* = <.05). Cohen’s *d* = .65 (medium effect).	Moderate
Douglas (2000)	Survey of Sexual Abuse (Tsai, Feldman-Summers & Edgar, 1979). Definition of sexual abuse limited to physical contact abuse occurring before the age of 16.	PSI-SF	T-tests and correlations	CSA mean stress score x Control mean stress score *t*(61) = 2.36, *p* = <.02. Cohen’s *d* could not be calculated.	Moderate
Ethier et al. (1995)	Psychosocial interview (including questions about CSA)	PSI	Correlation	*r* = .23 (negligent, not sig). *r* = .33 (control, *p* = <.01).	Weak
Harmer et al. (1999)	The Child Abuse & Trauma Scale (CATS) – sexual abuse scale	PSI	Correlation	*r* = .31 (*ns*, but maternal depression & social support subscales deleted for correlations to minimise collinearity with other measures included in the study)	Weak
Lang et al. (2010)	CTQ	PSI-SF	Correlations and multiple regression	PSI Defensive Responding: *B* = .12 (*ns*). PSI Parental Distress: *B* = .15 (*ns*). PSI Dysfunctional Interaction: *B* = .07 (*ns*).	Weak
Lutenbacher (2000)	Researcher questions: 1) Mothers were asked whether, before age 18, they had ever been touched in a sexual way against their wishes Was this action violent? Y/N 3 nominal categories: no SA, nonviolent SA and violent SA	Everyday Stressors Index (ESI)	Correlation	*r* = .22 (*ns*).	Weak
Mapp (2006)^a^	An adapted version of the questionnaire in Russell (1983): At least one contact or non-contact episode prior to the age of 18 where the perpetrator was at least 5 years older than the women or where force was used.	PSI	Path analysis	*r* = .14 (*ns*).	Moderate
Pazdera et al. (2013)^a^	An adapted version of the questionnaire in Russell (1983): At least one contact or non-contact episode prior to the age of 18 where the perpetrator was at least 5 years older than the women or where force was used.	PSI	Path analysis (mediation)	*r* = −.07 (*ns*).	Moderate
Pereira et al. (2012)	CTQ	PSI-SF	Correlation & ordinary least squares regression with bootstrapping	*r* = .13, *p* = <.05	Moderate
Renner et al. (2015)^a^	An adapted version of the questionnaire in Russell (1983): At least one contact or non-contact episode prior to the age of 18 where the perpetrator was at least 5 years older than the women or where force was used.	PSI Parent domain (5 of 7 subscales) PSI child domain (2 subscales only)	Latent Profile Analysis	CSA group reported higher mean scores on 5 PSI parent domain subscales. Cohen’s *d* = .22/.35/.37 for health/social isolation/depression subscales respectively. Restriction of role and attachment subscale *ns.*	Moderate
Schuetze and Eiden (2005)^a^	An adapted version of the questionnaire in Russell (1983): At least one contact or non-contact episode prior to the age of 18 where the perpetrator was at least 5 years older than the women or where force was used.	PSI	Structural Equation Modelling (SEM)	PSI Child Domain, *r* = .02 (*ns*). PSI Parent Domain, *r* = .18, *p* = <.01	Moderate
Wright et al. (2005)	Self-identification via a questionnaire, coded for severity by the researchers	PSI	Hierarchical regression analysis & mediator/moderator analysis	PSI Parent Domain mean scores markedly elevated on six of the seven subscales for CSA sample. PSI Child Domain x CSA severity, *r* = .01 (*ns*).	Weak

^a^Same primary data set used

*CSA* Childhood sexual abuse, *PSI-SF* Parental Stress Inventory – Short Form, *PSI* Parental Stress Inventory, *CTQ* Childhood Trauma Questionnaire

The Effective Public Health Practice Project (EPHPP) tool (Thomas et al. 2004) was used to assess the methodological quality of the studies identified as eligible for inclusion in the review. This tool identifies eight domains for studies to be rated on, the first six of which then combine into an overall quality rating for the study of “weak”, “moderate” or “strong”. To be classified as strong there must be four strong ratings across the six components with no weak ratings. To be classified as moderate there must be no more than one weak rating with less than four strong ratings. Finally, a weak rating is given for those studies with more than two weak ratings across the components. The EPHPP has been reported to have reasonable inter-rater agreement for the six domains and excellent inter-rater agreement for the overall final rating (Armijo-Olivo et al. 2012). The results of this appraisal are reported in Table 2. All studies were retained in the review following the quality appraisal which will be discussed further in the results section below.

## Results

### Study characteristics

Of the 14 eligible studies, four used the same primary data set for analysis (Mapp 2006; Pazdera et al. 2013; Renner et al. 2015; Schuetze and Eiden 2005). This means that there are 11 separate samples in this review with size of samples ranging from 44 to 483; a total of 1545 participants (see Table 1 for a summary of demographic characteristics). Of the 11 different samples, five employed a cross-sectional research design and two further studies included a case–control comparison group. The remaining four samples used a prospective design, measuring CSA at time point one and parenting variables at time point two. All studies recruited only mothers with six of the 11 samples from the USA, two from Canada, two from Australia and one from Scotland. Most studies recruited mothers from a non-clinical population (eight out of the 11 samples; *n* = 1391) mostly using a response to advert procedure and only three of the 11 samples were recruited from a clinical population (*n* = 154), including a mother and baby unit, a mental health outpatient clinic and a therapeutic community. Reporting on the ethnicity of participants varied: three studies did not report the ethnicity of participants; five of the 11 samples were mostly Caucasian participants and three samples reported a majority of African-American participants.

### Measures

#### Parenting stress

Eleven of the 14 studies (79%) used the Parenting Stress Index (PSI; Abidin 1995) or the Parenting Stress Index – Short Form (PSI-SF; Abidin 1995), see Table 2. One further study used several subscales of the PSI (Renner et al. 2015) and another study used a measure which included some items from the PSI (Barrett 2009). Only one study used an alternative measure, the Everyday Stress Index (Lutenbacher 2000). The frequent use of the PSI and the PSI-SF makes comparison between studies more viable.

#### CSA

In contrast, there was little homogeneity among studies regarding measurement of CSA (see Table 2). Two studies used the Childhood Trauma Questionnaire (CTQ; Bernstein and Fink 1998), but the remainder of the studies used either a different measurement tool such as the Child Abuse and Trauma Scale (CATS; Sanders and Becker-Lausen 1995) used in Harmer et al. (1999), or questions designed by the researchers.

Only six studies explicitly stated their definition of CSA (Alexander et al. 2000; Douglas 2000; Mapp 2006; Pazdera et al. 2013; Renner et al. 2015; Schuetze and Eiden 2005). Within these six studies, two limited their definition of CSA to contact abuse only (Alexander et al. 2000; Douglas 2000) and the remaining four, which used the same primary data set, included both contact and non-contact abuse. Additionally, the majority of studies used measures that simply measured the presence or absence of CSA. The exception to this is Wright et al. (2005) who initially asked mothers who had experienced CSA to respond to an advert for participants. Responses to the anonymised mailed questionnaire were then coded for severity by the researchers. In summary, the lack of consensual definitions and measurement of CSA makes comparison between studies difficult.

### Study quality

No studies were rated as strong in quality overall using the EPHPP tool (see Table 2). Eight were rated as moderate and six were rated as weak in quality, though several studies contained components that were rated as strong. Most of the studies were rated as moderate in the data collection section with three studies being rated as strong, mainly due to robust reporting of the reliability and validity of the measures used. Ten studies were rated as moderate on selection bias with the study sample considered to be at least somewhat likely to be representative of the target populations. However, four studies were rated weak mostly because participants self-referred into the study. A notable limitation in the majority of studies (*n* = 11) was the lack of description of possible confounding variables in either the methodological design or analysis of the studies. Most studies highlighted this issue later in the discussion section when suggesting possible explanations of their results, but very few address potential confounders earlier on.

### Direct associations between CSA and parenting stress

Seven of the 14 studies indicated a degree of direct association between experiencing CSA and later parenting stress, with six presenting statistically significant results (correlations ranged between *r* = .13 to .33; Cohen’s *d* ranged between .22 to .65) and one indicating the mean scores of the CSA group were markedly higher than the norms provided by Abidin (1995). Two of the 14 studies did not find any association between CSA and parenting stress and the remaining five studies suggest other variables may affect the relationship between CSA and parenting stress, such as locus of control and current stressors.

Two of the seven studies which found an association between CSA and parenting stress found a significant positive association between mothers who reported CSA and higher scores on the PSI-SF (Douglas 2000; Pereira et al. 2012). These two studies were from different samples. The remaining five studies reported significant associations between CSA and one subscale of the PSI (Buist and Janson 2001; Ethier et al. 1995; Renner et al. 2015; Schuetze and Eiden 2005; Wright et al. 2005), including the parenting domain (*n* = 4) and the optional life stress scale (*n* = 1).

Both the Douglas (2000) and Pereira et al.’s (2012) study were rated as moderate in quality. Douglas was only rated as weak on controlling for confounds as the study reported that the index group in this study were significantly more likely to be younger, live in a more deprived area and have experienced parental separation, divorce or death than the control group, yet these variables were not discussed in the method or controlled for in the analyses. The significant results in this study may therefore be accounted for by confounding variables such as these, with elevated stress reported by the index group possibly being associated with variables other than CSA per se. Alternatively, the significant results found in this study may be due to the very clear limits on the definition of CSA which was contact abuse only before the age of 16, whereas several other studies that report non-significant effects included non-contact sexual abuse (e.g. Mapp 2006). Arguably, lasting effects of CSA may be more likely following contact rather than non-contact sexual abuse, possibly accounting for the significant results in this study.

The significant results found in the Pereira et al. (2012) study may in part be due to the large sample size (*N* = 291) which may have been sufficient to detect subtle associations between CSA and parenting stress in the community sample and protect against type II errors. The study was rated as moderate in quality, only scoring one weak rating due to the cross-sectional study design. However, this study was rated as strong on data collection as it used measurement tools that have been shown to be both valid and reliable, the CTQ and the PSI-SF. The CTQ does include non-contact CSA, but the use of a standardised measure of childhood trauma which reports robust reliability (*α* = .91 for the whole scale, .94 for the CSA subscale in a community sample; Scher et al. 2001) may have enabled consistent reporting of experiences across participants.

Of the five studies that report associations between CSA and a subscale of the PSI, three were rated as moderate in quality and two were rated as weak. Buist and Janson’s (2001) study is of moderate quality overall, with a weak rating for the lack of description regarding control of confounding variables. They reported that the CSA group in their sample scored significantly higher on the optional life stress scale on the PSI than the comparison group (*d* = .65). As this is the only study to report the optional life stress subscale of the PSI it is difficult to make any assumptions about the significance of this finding. No significant difference was reported between the CSA group and the comparison group on either the parent or child domain of the PSI which may be due to a lack of power as the sample size was relatively small (*N* = 45; CSA group *n* = 23, comparison group *n* = 22) which increases the possibility of type II error.

Renner et al. (2015) found that women reporting CSA had slightly higher mean scores on all five subscales of the PSI parenting domain they included in their study when compared to women not reporting CSA. Effect sizes were calculated for these subscales and three were found to show a small effect (see Table 2). Additionally, Schuetze and Eiden (2005) reported that CSA was significantly associated with parenting stress on the parent domain of the PSI, but not significantly associated with the child domain. Both these studies used the same primary data set and are of moderate quality, which suggests the results reported may reliably indicate that there is a degree of association between CSA and later parenting stress on the parent domain of the PSI for the participants in this study, which were drawn from a community sample.

Both Ethier et al. (1995) and Wright et al. (2005) were rated as weak on the quality assessment tool, though both reported associations between CSA and scores on the parenting domain of the PSI. Ethier et al. explored issues pertaining to motherhood for negligent mothers, with parental negligence defined as “a serious omission from the parent who endangers the child’s development” (p. 622). All mothers in this group had been implicated in severe maltreatment and were found to experience significantly higher levels of stress than the control group. Both the index and comparison groups contained mothers with histories of CSA and Ethier et al. found that total sexual abuse was significantly associated with stress on the parent domain of the PSI for both the index and comparison group. However, only the mothers in the control group were found to have significant associations with CSA on the total stress score. One possible explanation for this is that the index group may have more current daily stresses than the control group, given their alleged maltreatment of their children. The effects of CSA therefore appear more salient for the control group who may not have such difficult situations to contend with.

Finally, with regard to direct associations between CSA and later parenting stress, Wright et al. (2005) found that the mean scores for mother’s reporting CSA on the parent subscales of the PSI were markedly higher on six out of seven subscales compared to the normative sample from Abidin (1995). Again, this provides further support for an association between the parent domain of the PSI in particular and historical CSA. However, this study was predominantly weak in quality, particularly with regard to selection bias and research design, as participants had responded to an advert asking for mothers who had experienced CSA. This self-selection bias may have skewed the results making the sample in the study not representative of the population of people who have experienced CSA.

Two studies reported no association between CSA and later parenting stress. Alexander et al. (2000) did not find a significant main effect of CSA on parenting stress. However this study was rated as weak in quality with a cross-sectional design, possible selection bias with recruitment relying on response to advert and lack of control for confounders. The second study, Barrett (2009), was rated as moderate in quality and had the largest sample in this review (*N* = 483). Barrett reported the mean of the CSA group was not significantly different from the control group on the measure of parenting stress used and CSA did not reach significance in the regression analysis (see Table 2). It is possible that the use of non-formal measurement tools affected the results obtained and this component was rated as weak on the EPHPP. For example, the CSA measure was: “has a stranger, acquaintance, date or relative ever tried or succeeded in doing something sexual to you against your wishes?” (p. 496) with affirmative responses followed up with a question regarding age of occurrence. This may also mean that the abuse group included participants for whom the abuse may not have been as severe as other studies which used a more stringent measure of CSA such as Douglas (2000) who defined CSA as women with a history of contact child sexual abuse before the age of 16. Idiosyncratic measurement of CSA is not unusual throughout the studies in this review, but for parenting stress other studies used a validated measure whereas Barrett did not, opting instead for a scale from a women’s employment study which was conducted in the USA, that “included items from the PSI” (p. 497). It is possible this measure was not a valid or reliable measure of parenting stress which may have skewed the results in the study. Furthermore, despite the Barrett study having a large sample, the percentage of CSA survivors in this sample was actually the smallest out of all the studies included in this review (11%, see Table 1). This increases the possibility of a type II error as it may seem as though there was no effect of CSA on parenting stress when the sample size of CSA survivors was not sufficient to detect any effect.

Only two studies limited their inclusion criteria to contact CSA only: Alexander et al. (2000) who did not find any association between CSA and later parenting stress and Douglas (2000) who found that mothers in their CSA group reported significantly more stress overall than their comparison group. This difference in results may be due to the methodological quality of the studies: Alexander et al. was rated as weak in quality and Douglas was of moderate quality. An alternative explanation may be that the Douglas study used a clinical sample from a mental health outpatient clinic where participants may be experiencing elevated stress due to their mental health difficulties rather than due to parenting per se, whereas Alexander et al. recruited from the community where there may be less variation in the data. Lastly, the Douglas study contained a greater proportion of CSA survivors (54%) compared to the Alexander et al. study (21%) which may have enhanced the potential of identifying an association between CSA and parenting stress.

In summary, there is no strong, consistent evidence of a direct association between CSA and later parenting stress. However, the results suggest that contact-only CSA may produce a significant association with parenting stress and that studies including both contact and non-contact CSA may need larger sample sizes to detect smaller effects. Several studies suggest elevated stress on the parenting domain of the PSI but not the child domain which suggests participants were more likely to attribute parenting stress to their own characteristics rather than the characteristics of the child.

### Possible mediating factors between CSA and parenting stress

#### Depression

Depression was highlighted in 10 of the studies as having a significant association with both CSA and parenting stress. The results of eight of these studies suggest there may be a potential indirect path from CSA to parenting stress through current level of depression (Buist and Janson 2001; Douglas 2000; Ethier et al. 1995; Lutenbacher 2000; Mapp 2006; Pazdera et al. 2013; Schuetze and Eiden 2005; Wright et al. 2005). Five of these studies were of moderate quality and three were weak in quality. The other two studies, both rated as weak in quality, found a significant association between depression and parental stress, though the association between CSA and depression was not significant (Harmer et al. 1999; Lang et al. 2010). Of the eight studies which found significant associations between CSA, level of depression and parenting stress, three of these used the same primary data set (Mapp 2006; Pazdera et al. 2013; Schuetze and Eiden 2005) and hence the same measure of depression; the Center for Epidemiologic Studies Depression Scale (CES-D; Radloff 1977). This scale was also used in the Lutenbacher (2000) and Wright et al. (2005) study while the Beck Depression Inventory (BDI; Beck et al. 1961) was used in both the Buist and Janson (2001) and the Ethier et al. (1995) study. Buist and Janson also used the Hamilton Rating Scale for Depression (HDRS) and Douglas (2000) found a significant association between the depression subscale on the General Health Questionnaire (GHQ-28) and parenting stress for both the CSA group and the comparison group. The results of these eight studies, which used different but reliable methods of measuring depression, suggest depression is a significant factor in the association between CSA and parenting stress.

With regard to the two studies which found a significant association between depression and parental stress, yet not between CSA and depression, Lang et al. (2010) found depression was significantly negatively related to defensive responding and parental distress on the PSI-SF at one year postpartum. This means that participants reported less parental distress than they may actually be experiencing. However, conclusions from Lang et al. should perhaps be interpreted with some caution because the study was of weak quality overall and retained only 31 out of 44 participants for the postpartum follow-up. Such attrition may result in a biased sample at follow-up and this small sample size is not particularly representative, making analysis susceptible to type II errors. Similarly, Harmer et al. (1999) was rated as methodologically weak and reports that some mothers chose not to complete all measures. The number of participants per measure ranged from 39 to 46 and five participants chose to complete the measures with the assistance of a researcher, which increases possibility of demand characteristics. Furthermore, approximately half of the remaining participants had missed occasional questions when they returned the measures, which the researcher subsequently supported them to complete, again elevating the risk of bias.

Five studies conducted mediation analysis with their data (Mapp 2006; Pazdera et al. 2013; Pereira et al. 2012; Schuetze and Eiden 2005; Wright et al. 2005) though only three report CSA and parenting stress as predictor and outcome variables and depression as a mediator, which are the three studies which use the same primary data set (Mapp 2006; Pazdera et al. 2013; Schuetze and Eiden 2005). The other two studies report mediation using different outcome variables including maternal sensitivity (Pereira et al. 2012) and resilience domains (Wright et al. 2005). Mapp (2006) reported the results of a path analysis which indicated the only significant route from CSA to elevated scores on the PSI was through the level of current depression. This study also noted locus of control impacted scores on the PSI both directly (*r* = .47) and through depression (*r* = .45). Both Pazdera et al. (2013) and Schuetze and Eiden (2005) included other variables in their mediation models which precludes clear conclusions being made regarding whether depression mediates the association between CSA and parenting stress. Pazdera et al. (2013) conducted a multiple mediation model which included CSA as predictor, parenting sense of competence and depression as mediators, and parenting stress and maltreatment behaviour as outcome variables. They reported the fit of the model to the data was relatively poor (*χ* ^*2*^(7) = 36.17, *p* = <.001). Similarly, Schuetze and Eiden (2005) found that partner violence, along with depression, mediated the association between CSA and the outcome variables which were parenting attitudes (including both parenting stress and parenting competence) and punitive discipline. However, the model did not fit the data particularly well (*χ* ^2^(21) = 38.17, *p* = <.05). These results suggest variables other than depression may impact the association between CSA and parenting stress, though investigation of these relationships was only conducted in studies which used the same primary data, demonstrating a need to replicate these findings in different samples.

As indicated above, the studies included in this review measured a number of other variables alongside CSA, depression and parenting stress. There was little homogeneity between studies in terms of variables measured, but several studies indicated significant associations with other factors. Positive belief systems were found to be negatively associated with parenting stress in six studies (Buist and Janson 2001; Lutenbacher 2000; Mapp 2006; Pazdera et al. 2013; Renner et al. 2015; Schuetze and Eiden 2005). For example, higher self-esteem was negatively associated with stress in the Lutenbacher (2000) study (*r* = −.48, *p* = <.001) and higher scores on parenting satisfaction and self-efficacy were associated with lower scores on parenting stress in Pazdera et al. (2013) and Schuetze and Eiden (2005) (associations ranged between −.41 to −.68, *p* = <.01). Similarly, higher social support and/or relationship satisfaction were associated with lower parenting stress for CSA survivors in three studies (Alexander et al. 2000; Harmer et al. 1999 and Wright et al. 2005). Such factors may therefore be potential mediators or moderators of the relationship between CSA and parenting stress, though were not tested as such in the studies.

Seven studies included measures of various other forms of childhood maltreatment, including neglect and physical and emotional abuse (Alexander et al. 2000; Barrett 2009; Ethier et al. 1995; Harmer et al. 1999; Lang et al. 2010; Lutenbacher 2000; Pereira et al. 2012). Different types of childhood maltreatment were associated with each other in most of these studies and parenting stress was associated with the experience of childhood physical abuse in four studies (Barrett 2009; Ethier et al. 1995; Lang et al. 2010; Pereira et al. 2012), with neglect/negative home environment in two studies (Ethier et al. 1995; Harmer et al. 1999) and emotional abuse in two studies (Lang et al. 2010 and Pereira et al. 2012). Furthermore, current partner violence was also associated with stress in two studies which included a measure of this (Lutenbacher 2000; Schuetze and Eiden 2005), though was only associated with CSA in Schuetze and Eiden (2005).

Finally, only six of the 14 studies reported characteristics of the CSA experienced by their participants (Alexander et al. 2000; Buist and Janson 2001; Douglas 2000; Lutenbacher 2000; Schuetze and Eiden 2005; Wright et al. 2005). Despite the range of experiences within the categorisation of CSA, only Douglas (2000) reported analyses using these different types of experience, finding no significant difference between scores on the PSI for intra and extra-familial abuse. No studies included analysis of other potential moderators, such as age or severity of abuse, so conclusions regarding different aspects of CSA and the effects on later parenting stress could not therefore be inferred.

## Discussion

In summary, seven studies suggest there is a direct association between CSA and parenting stress. Depression was identified as a possible mediator between CSA and parenting stress in ten studies, indicating the existence of an indirect pathway from CSA through depression to parenting stress. Studies also suggested that other potential variables may affect the association between CSA and parenting stress, such as co-occurring childhood maltreatment, sources of support and internal belief systems. The lack of consensual definition of CSA makes comparison between studies difficult as what is categorised as CSA in one study may not be classed as such in another, for example, contact versus non-contact CSA. Finally, it appears that the association between CSA and parenting stress may be influenced by both sample size and reliable measurement tools, with larger sample sizes and psychometrically validated measures producing more significant associations between these two variables.

An association between historical experiences of CSA and later parenting stress was found in both clinical (*n* = 2) and non-clinical (*n* = 5) samples. However, four of the seven studies which found a direct association between CSA and parenting stress reported this was significant only for the parenting domain of the PSI. One explanation for this finding is that early experiences of CSA may lead to the development of internalising disorders such as depression and anxiety (Sachs-Ericsson et al. 2010) and lower self-esteem (Schuck and Widom 2001). This means individuals are more likely to make negative appraisals of themselves and their abilities, perhaps resulting in attribution of stress to their own characteristics rather than their child.

Evidence of the potentially mediating role of depression in the CSA and parenting stress relationship is supportive of past research which indicates that people who experience CSA are vulnerable to developing depression (Wangel et al. 2016) and that the experience of depression is associated with increased parental stress (Zajicek-Farber et al. 2012). CSA may increase the risk of experiencing depression, which then affects the experience of parenting, or in turn CSA may cause difficulties in parenting which then may give rise to feelings of depression. However, it is important to consider the role of reporting bias in understanding these relationships, as the presence of depression itself may lead to more negative responses on self-report questionnaires (Bistricky et al. 2014). Participants may therefore be managing the parenting role adequately, but depression affects their self-judgement and leads them to negatively appraise their abilities.

The results of this review suggest contact abuse has a stronger relationship with later parenting stress than non-contact abuse. There is limited previous research on the differential effects of contact versus non-contact CSA (Landolt et al. 2016), but hypothetically contact abuse is a more invasive violation than non-contact abuse, resulting in greater negative sequelae. For example, survivors of more severe forms of abuse have been reported to experience more symptoms of depression than those who experienced less severe abuse (Seltmann 2013). However, it is important to continue to investigate non-contact CSA as the results of this review suggest effects can be detected between CSA and parenting stress if the sample is large enough, suggesting weaker but nonetheless significant findings.

It is also important to consider other aspects of abuse that may determine the effects that the experience has on parenting and other outcomes. For example, recent research regarding the effects of CSA on a child’s developing brain suggests the age maltreatment occurs may have a significant impact on the negative sequelae experienced, with the younger the age of onset, the more impactful the maltreatment. It is suggested that early exposure to adversity sensitises parts of the brain, most notably the amygdala and the hippocampus, to later stress (Teicher and Samson 2016). It may be that those studies which found stronger associations between CSA and later parenting stress included participants who experienced CSA at an earlier age than the other studies which did not find significant associations. Similarly, research has found that individuals experiencing CSA before age 12 are more likely to report higher rates of depression than individuals abused after this age (Schoedl et al. 2010). However, the studies in this systematic review grouped experience of CSA together as a homogenous group with only six reporting any characteristics of the CSA participants and only one study (Douglas 2000) reporting analyses between CSA characteristics, finding no significant difference between scores on the PSI for intra and extra-familial abuse. More research needs to be conducted to explore such moderators of the association between CSA and parenting stress.

The relationship between historical CSA and later parenting stress is complex and many additional historical and contemporary factors may influence this association. For example, consistent with previous research (e.g. Hughes and Cossar 2015), seven of the studies in this review found significant associations between other types of childhood maltreatment and parenting stress, including physical abuse, neglect and emotional abuse. The studies in this review also found that other mediators were significant in their analysis of the relationship between CSA and parenting stress, including locus of control, parenting sense of competence and current partner violence. These findings suggest that feelings of disempowerment and being unable to effect change may be significant mediators of the association between CSA and parenting stress. This may result in internalising disorders and depressive symptoms, as described above (Sachs-Ericsson et al. 2010) which in turn may influence parenting stress. Insecure attachment is another potentially important mediator that was not examined by the studies included in this review. Research links early life trauma with insecure attachment (e.g. Murphy et al. 2014) and research also suggests an association between attachment insecurity and parenting stress (Kwako et al. 2010).

Conversely, protective factors, such as positive belief systems and partner/social support, were found to be negatively associated with parenting stress in this review which supports previous research in this area (e.g. Zvara et al. 2015). A secure attachment style may also be a protective factor against parenting stress and a secure attachment may contribute to the development of resilience (Rutten et al. 2013) which is an important factor to consider regarding the development of negative sequelae.

### Clinical implications

The results of this review have implications for health and social services working with mothers who have experienced CSA. Firstly, postnatal services should be mindful of potential contributing factors to new mothers’ difficulties, such as previous CSA and the effect this may have on their parenting abilities. Mothers who experience difficulties beyond those expected due to normal adjustment should perhaps receive a more comprehensive assessment, which includes factors relating to their own early life experiences. Secondly, professionals in postnatal services, such as midwives and health visitors should be trained how to ask service users about early life experiences. For example, Read et al. (2007) gives clear guidelines for how mental health services should ask about trauma which might also be useful for staff working in postnatal services. For example, he recommends introducing such questioning as “I’m going to ask you about some unpleasant things that happen to some people in childhood. We ask because sometimes it helps throw light on difficulties later in life” (p. 106). Mothers could then be signposted to appropriate mental health or therapy services if they wanted further support. Thirdly, for mothers who access services later due to depression and/or stress, robust formulation should consider their early life experiences (Read 2006) and link this to their presenting problems. This would ofer a clear, theoretically based explanation of the mothers’ difficulties to facilitate understanding and determine potential areas for intervention. Offering interventions for treating depression, such as Cognitive Behavioural Therapy (CBT), may reduce levels of depression and indirectly impact on levels of stress.

Finally, research suggests that elevated parenting stress can have a detrimental impact on the parent–child relationship and potentially result in negative outcomes for the child (Soltis et al. 2015). Parenting stress can be addressed directly through parenting programmes such as The Incredible Years programme (Webster-Stratton 2006) which aims to improve parenting abilities and subsequently child functioning. Research on parenting programme indicates parents experience reductions in both stress and depression following completion of the intervention (Bennett et al. 2013), which has a positive consequence on child outcomes.

### Strengths and limitations of the review

This systematic review is the first to explore the association between CSA and later parenting stress and several strengths are noted. Firstly, the review was conducted transparently following the PRISMA Statement (Liberati et al. 2009) which enables readers to assess the quality of the review and replicate the search. Secondly, the method employed was thorough, searching seven key databases using comprehensive search terms. Finally, studies included were assessed for quality which allowed critical appraisal of the findings of each study and the strength of the evidence overall could be assessed.

However, the absence of a shared definition of CSA and the lack of homogeneity regarding measurement of CSA limits the ability to draw firm conclusions about the association between CSA and later parenting stress. Haugaard (2000) suggests that a definitive definition of childhood sexual abuse is challenging as perceptions of what constitutes CSA may vary between clinicians, researchers and legal systems. This problem is pervasive as Barth et al. (2013) conducted a systematic review and meta-analysis on the prevalence rates of CSA worldwide and found notable diversity in how CSA was defined between studies. Furthermore, most studies in this review used different measures of CSA and many used idiosyncratic questions developed by the researcher which makes the reliability of the data questionable. Reporting bias and underreporting in particular are significant problems in research investigating sensitive topics like abuse and parenting, and these problems are further compounded by poor measurement instruments.

The review also focused on the effects of CSA on later parenting stress and excluded other types of childhood abuse from the main analyses. This limits the inferences that can be made from this review, and conclusions cannot be generalised to other types of childhood abuse. As can be seen in the results section, other forms of childhood abuse that were measured in these studies were found to have significant associations with both CSA and parenting stress. Including these along with CSA may have allowed a more comprehensive review of the effects of any childhood maltreatment on later parenting stress. Additionally, all the studies in this review focused on women and excluded men. Results therefore cannot be generalised to men which highlights a gap in understanding how CSA may affect parenting stress for fathers. Furthermore, approximately half of the studies in the current review included predominantly Caucasian participants and half included predominantly African-American participants. It is worthy of note that the two studies that reported a significant direct association between CSA and later parenting stress (Douglas 2000; Pereira et al. 2012) used mostly Caucasian populations. Under reporting of CSA may be a problem in some populations which may affect results, particularly in studies using comparison groups, by including participants who had experienced CSA in comparison groups rather than the CSA groups.

Finally, the inclusion criteria for this review means some potential articles may have been excluded, such as grey literature and studies published in languages other than English.

### Directions for future research

A number of potential avenues for future research have been highlighted by this review. Firstly, the most pressing task for further research in CSA is to agree definitions and validate measures for this population. Secondly, the age at which CSA was experienced should be explored as a moderator of the association between CSA and later parenting stress with a tentative hypothesis being the younger the age of CSA onset, the more likely later parenting stress will be elevated. Other moderators of the relationship between CSA and parenting stress, such as severity and type of perpetrator and current life stressors including partner violence, should also be explored, as the results of this review indicate limited investigation of these aspects. Thirdly, the role of further mediators and protective factors in the association between CSA and parenting stress, such as attachment, resilience, locus of control and parenting sense of competence, should be explored further as this may provide additional information regarding the relationship between CSA and parenting stress. Finally, the gap in research pertaining to the effects of CSA on fathers should be addressed to explore if there is an association between CSA and later parenting stress for men.

## Summary and conclusions

This systematic review found significant associations between CSA and later parenting stress, though the results suggest that this effect is mediated by depression. Other variables may also mediate or moderate this relationship, such as attachment or abuse severity, but their role in the CSA and parenting stress relationship needs to be more fully explored in future research. Clinical implications arising from this review include the importance of training staff to ask about early life experiences in mothers who are struggling and the need to offer interventions to address parenting stress.
